# Supplementary material for: Optical Biopsy of Dysplasia in Barrett’s Oesophagus Assisted by Artificial Intelligence
Source: Cancers (Basel). 2023 Mar 24;15(7):1950. doi: 10.3390/cancers15071950 (PMC10093622; doi:10.3390/cancers15071950)
Supplement: Supplementary file 1 [file cancers-15-01950-s001.zip › cancers-2229688-supplementary.pdf]

SUPPLEMENTARY MATERIAL S1: in vivo EC imaging procedure

Endoscopic examination of BE was carried out using the GIF-H290 EC model, which records 25 frames per second. Three operators (FP, JW, WN) with extensive experience in endoscopic BE assessment performed all EC procedures in 52 patients (Supplementary Table S1). Endoscopic procedures through EC were all conducted in the same sequential manner.

Supplementary Table S1 Baseline characteristics of the EC procedures

**Sex of patients**	Female	18 (34.6%)
	Male	34 (65.4%)
**Age of patients**	Years	66.1 (±11.5)
**Indication for gastroscopy**	BE surveillance	37 (71.1%)
	EMR procedure	15 (28.9%)
**Operators**	Operator 1	29 (55.8%)
	Operator 2	20 (38.5%)
	Operator 3	3 (5.8%)
**EC imaged site with targeted biopsy**	Lesion	52 (66.7%)
	Non-aberrant mucosa	26 (33.3%)
**Histopathology result**	Metaplasia	36 (46.2%)
	Indefinite for dysplasia	4 (5.1%)
	Low grade dysplasia	7 (9.0%)
	High grade dysplasia	12 (15.4%)
	Carcinoma	19 (24.4%)

EC (endocytoscopy); BE (Barrett's esophagus), EMR (endoscopic mucosal resection)

Macroscopic assessment

The endoscopic evaluation of the mucosa was initiated using HD-WLE and Narrow Band Imaging (NBI) to assess the Barrett's segment and screen for the presence of a lesion. The extent of the Barrett's segment was graded according to the Prague C&M classification. If a macroscopic lesion was observed, its presence was documented according to clinical practice including its distance to tooth line (upper and lower border) and its circumferential location (1 to 12 o' clock).

Endocytoscopic assessment

Prior to endocytoscopic imaging, we first sprayed N-acetylcysteine along the tissue surface to remove the mucus. Then, we stained the mucosa with a mixture of 0.05% crystal violet (CV) and 1% methylene blue (MB) through a single-use spray catheter (PW 205V, Olympus, Tokyo, Japan). CV is thought to stain the cytoplasm of the cells pink whereas MB stains the cellular nuclei. We employed an imaging window of 3.5 minutes to record EC frames. An absorption time of 90 seconds was taken into account to stain the tissue, and the staining resolves after five minutes since its application as the cells then progressively detach from the superficial mucosal layer. In order to allow endocytoscopic observation, lens-mucosa contact needs to be established by gently pushing the distal tip of the endocytoscope to the esophageal wall.

Endocytoscopic recording

Dependent on the presence of a lesion in the Barrett's segment, we employed a specific strategy for endocytoscopic recording. If no macroscopic lesion was present during a surveillance examination, EC imaging was performed at the circular border of the Z-line at 3, 6, 9 and/or 12 o' clock which would also be sampled according to the Seattle protocol. If a lesion was present, we first videotaped an adjacent, macroscopically non-aberrant spot and afterwards the lesion to potentially facilitate an endocytoscopic comparison within a Barrett segment between non-suspected and suspected tissue. In case EC was performed during an endoscopic removal therapy of an established Barrett's associated

neoplastic lesion, argon plasma coagulation spots were applied to mark the borders of the lesion prior to EC observation. The coagulation spots were used to image adjacent macroscopically non-suspected and suspected BE tissue. Each EC video was stored digitally in the RVC Clinical Assistant software used in the UMCG to record GI endoscopy imagery.

Tissue sampling

To establish a correlation between EC and histopathology, we obtained at least two biopsies of the imaged site and placed separately in their own dedicated jar. Targeted mucosal specimens were acquired directly after EC imaging. As the working channel is next to the Endocytoscopic lens at the distal tip of the endoscope, targeted biopsies are possible. Histopathology analysis of the samples was done by experienced GI pathologists to check for the presence of metaplasia or neoplasia.

SUPPLEMENTARY MATERIAL S2: **EC image classification**

1. Literature search to identify potential EC features in BE

Before we initiated the acquisition of endocytoscopic BE videos, we conducted a literature study to identify potential EC features in BE. The endocytoscopic experience in BE is however limited. Pohl et al. concluded in 2009 that in vivo EC application (x450 and x1100 magnification) in BE was infeasible [1]. In 2013, an ex vivo endocytoscopic BE classification system (x1100 magnification) that included detailed cellular and nuclear features was published by Tomizawa et al. [2]. In contrast to EC, various studies report ultra-magnified imaging of BE through confocal laser endomicroscopy (CLE) [3], and in our center we also use CLE for BE assessment in a clinical setting. Although CLE images are in grayscale and EC images are in a red-to-blue spectrum, we hypothesized that both modalities would present largely similar tissue features. Considering the literature we found, we decided to prospectively score for architectural features according to a validated CLE classification system [4-6], and for cellular and nuclear features according to the study by Tomizawa et al. [2]. For the interpretation of microvasculature that could potentially be observed during EC, we used a simplified description by Kara et al. [7].

2. Prospective scoring of endocytoscopic BE features during in vivo procedures

Directly after each EC procedure, the operator had to determine whether the in vivo EC video provided sufficient quality to distinguish potential endocytoscopic BE features: high-quality or low-quality videos. This assessment was established in consideration of the image resolution and degree of staining (Supplementary Table S2).

Supplementary Table S2 The comparison of staining quality and image resolution between high-quality videos (N = 61) and low-quality videos (N = 17)

		**High-quality videos**	**Low-quality videos**
**Quality of staining***	Poor	17 (21.8%)	16 (20.5%)
	Good or excellent	44 (56.4%)	1 (1.2%)
**Resolution of image***	Poor resolution	5 (6.4%)	16 (20.5%)
	Moderate resolution	15 (19.2%)	0 (0%)
	Good resolution	41 (52.6%)	1 (1.2%)
**Histopathology**	Metaplasia	31 (39.7%)	5 (6.4%)
	Indefinite for dysplasia	2 (2.6%)	2 (2.6%)
	Low grade dysplasia		
	High grade dysplasia	6 (7.7%)	1 (1.2%)
	Carcinoma	10 (12.8%)	2 (2.6%)
		12 (15.4%)	7 (9.0%)

High-quality EC videos had generally a better resolution and quality of staining than the low-quality videos. *$P<0.05$.

High-quality EC videos were then scored for the presence of the potential features. The operators were able to view the features in example images, and then checked boxes for the features they had observed during the in vivo procedure. Supplementary Table S3 provides an overview of the number of aberrant features that could be observed during HQ EC videos in BE according to the performing operator. We excluded the high-quality videos that had indefinite for dysplasia in their targeted samples (N = 2) from analysis.

Supplementary Table S3 Number of observations of endocytoscopic features that were recorded during in vivo endocytoscopic BE examinations by the performing operators

		High-quality EC videos of histologically verified non-dysplastic BE (N = 31)	High-quality EC videos of histologically verified dysplastic BE (N = 28)
**Aberrant vascular pattern***	No	23 (39%)	4 (6.8%)
	Yes	7 (11.9%)	22 (37.2%)
**Aberrant vascular morphology***	No	28 (47.5%)	11 (18.6%)
	Yes	3 (5.1%)	17 (28.8%)
**Aberrant size/shape of the glands ****	No	24 (40.7%)	13 (22.0%)
	Yes	7 (11.9%)	15 (25.4%)
**Aberrant arrangement of the glands***	No	3 (5.1%)	8 (13.6%)
	Yes	28 (47.5%)	20 (33.9%)
**Aberrant cellular features**	No	30 (50.8%)	26 (44.1%)
	Yes	1 (1.7%)	2 (3.4%)
**Aberrant nuclear features***	No	28 (47.5%)	13 (22.0%)
	Yes	3 (5.1%)	15 (25.4%)

*$P<0.001$; **$P=0.014$

3. Design of the endocytoscopic BE classification system

After prospectively acquiring all EC videos, there was a final meeting including a BE expert pathologist (AK) to achieve consensus regarding the endocytoscopic features that could be observed in non-dysplastic and dysplastic Barrett's tissue. Based on the in vivo observations, we decided to include the vascular pattern and morphology, architectural features, and nuclear features in the classification system. As cellular features were rarely observed, we decided to exclude these from the classification system. Due to the relatively small sample size of the EC videos, we chose to cluster the features of the neoplastic changes in BE together (LGD, HGD, EAC). Moreover, discordance in classifying various grades of dysplasia exists even among expert pathologists.[8] In addition, it appeared to be difficult to correlate the EC morphology exactly to the various stages of dysplasia when these were present in one mucosal specimen. We decided to exclude the videos that had targeted biopsies showing indefinite for dysplasia. Thus, we eventually designed a binary endocytoscopic classification of Barrett's tissue of which we considered to be relevant for clinical translation of EC-guided biopsies: EC metaplasia and EC neoplasia. EC metaplasia represents metaplastic columnar epithelium of the fundic-type, cardia-type, or interstinal-type that have been histologically verified. EC neoplasia represents histologically verified LGD, HGD, and carcinoma. For an overview of the features, see Supplementary Table S4.

Supplementary Table S4 Proposed in vivo endocytoscopic BE classification system

	**EC Metaplasia**	**EC Neoplasia**
**Architectural features**	Distinct tubular structures of uniform shape and size and oval to round lumina;<br>Well-organized arrangement.	Variable size and shape of the glands with shortening or narrowing lumina;<br>Distorted arrangement.
**Cytologic features**	Nuclei are either not visible or as small, round blue dots;<br>Goblet cells as white, transparent dots.	Nuclei can be visible as blurred, aggregated dots.
**Vascular features**	Long, branching vessels around the glands	Small, tortuous vessels randomly distributed.

4. Development of the online training and testing modules

We developed a training program and two test sets as online modules in REDCap (Research Electronic Data Capture). The training program was designed as a stepwise introduction to the EC procedure and the endocytoscopic BE features. During the online training, we provided two images to describe the

vascular features, and four images to describe the architectural and nuclear features with corresponding H&E specimens. Arrows and circles were used to point out the corresponding features in the EC images. Afterwards, participants had access to six videos of 20-30 seconds with corresponding H&E specimens. Two of these videos were narrated in Dutch to describe the features that could be observed in the videos (the same videos narrated in English are separately uploaded as Supplementary Material to this manuscript). All of the images and videos were provided in a 1:1 ratio for EC metaplasia and EC neoplasia. For this article, we created a separate link to access the online training program: https://redcap.link/Endocyto_AI_training.

To assess the effect of training, the first test set was completed by the online participant before and after training, with the same images in different sequences (Supplementary Figure S1). After a two-week interval, an invitation for the second test set was sent to assess the effect of wash-out. The second test set contained a different set of images than the first test set. Each test set contained 30 images: 15 EC metaplasia and 15 EC neoplasia. The online testing and training for the participants were accessible via https://redcap.link/Endocyto_testing_and_training.

During the assessments of the EC frames, participants were blinded to patient history, overview inspection under HD WLE or NBI, and histopathological diagnoses. They were unaware of the proportion of neoplastic images in the test sets. During the test sets, all participants were unable to review previously seen images or to change their answers, and they did not receive feedback on their given answers at the end of the test. The baseline characteristics of the participants that were included for the participation in the online modules are listed in Supplementary Table S5.

Supplementary Table S5 Baseline characteristics of the participants that were included for the online modules.

	Cohort 1 (N=10)	Cohort 2 (N=12)
Gastroenterology residents	5 (50%)	0
Gastroenterologists with expertise in BE from the Dutch BE expert centers	5 (50%)	0
Gastroenterologists from academic center	0	9 (75%)
Gastroenterologists from community hospitals	0	3 (25%)
Experience in Endocytoscopy	0	0
Experience in AI	2 (20%)	0

SUPPLEMENTARY MATERIAL S3: training and testing of the AI algorithm

To develop an atlas of endocytoscopic BE images, EC videos (N = 78) were reviewed (Supplementary Fig. S1). After exclusion of videos (N = 19) (see Supplementary Table S2), 83,277 frames were extracted from 59 videos from 37 patients to construct data sets. Patients were split on a per-patient-basis into either the training set or the test sets. Some patients contributed EC metaplasia as well as EC neoplasia frames to a data set.

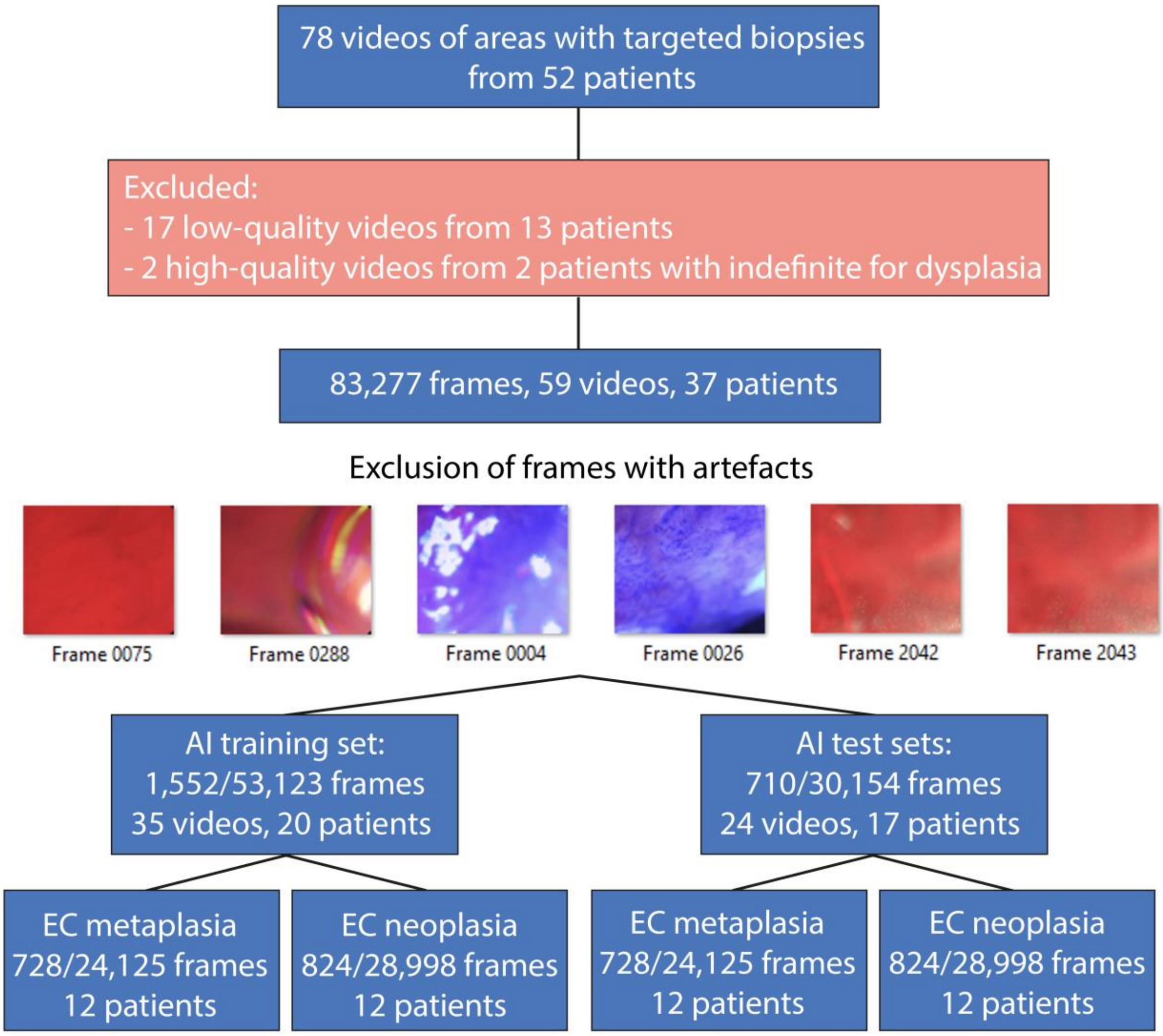

Supplementary Figure S1 Overview of the review and selection of the EC frames for training and testing the AI model.

SUPPLEMENTARY MATERIAL S4: experiences of the endoscopists regarding the online modules

The endoscopists scored the online training a 7.8 (±1.4) [0-10 scale] for quality and they spent 14.9 (±7.0) minutes to it. Test sets did not differ in quality of images (Supplementary Table S6), or in the experienced level of difficulty (7.6 (±1.3) vs 6.8 (±1.1), *P*=0.174) [0-10 scale]. Participants spent equal time to test set 1A (9.6 (±10.2) minutes), test set 1B (7.5 (±1.6) minutes), and test set 2 (7.6 (±1.3) minutes) (*P*=0.256).

Supplementary Table S6 Rating of the quality of the images used in the two test sets according to the online participants

	**Test set 1**	**Test set 2**
**Bad quality**	0	1 (5%)
**Moderate quality**	6 (30%)	8 (40%)
**Good quality**	4 (20%)	1 (5%)

Compared to the proportion of true diagnoses with high confidence before training (14.7%), the indicated proportion increased after training (32.3%, *P*=0.001). At follow-up, the proportion of true diagnoses with high confidence (20.3%) was lower than directly after training again (*P*=0.001).

SUPPLEMENTARY MATERIAL S5: experiences of the unassisted and AI-assisted gastroenterologists regarding the online modules

As depicted in Supplementary Table S7, the unassisted and AI-assisted gastroenterologists rated the quality of the online training program equally (7.1 (±1.2) vs 7.0 (±1.1), *P*=0.882), felt equally prepared to do the testing after the training (7.1 (±1.2) vs 6.2 (±0.9), *P*=0.140), and spent an equal amount of time to the training (15 (12-19.3) vs 16.0 [13-37.5] *P*=0.429).

Supplementary Table S7 Experiences regarding the online training module and the time spent to the online training

	Unassisted gastroenterologists (N = 6)	AI-assisted gastroenterologists (N = 6)	*P*-value
Quality rating [0-10 scale]	7.1 (±1.2)	7.0 (±1.1)	0.882
Rating of feeling prepared after training [0-10 scale]	7.1 (±1.2)	6.2 (±0.9)	0.140
Time spent (minutes)	15 (12-19.3)	16.0 (13-37.5)	0.429

As summarized in Supplementary Table S8, the quality of the images that were included in the two test sets were assessed equally by both the unassisted gastroenterologists (*P*=0.574) and the AI-assisted gastroenterologists (*P*=0.545). Likewise, the two test sets did not differ in level of difficulty according to the gastroenterologists from both groups (7.1 (±1.5) vs 7.8 (±1.6), *P*=0.478 and 6.3 (±2.3) vs 7.6 (±0.9), *P*=0.215).

Supplementary Table S8 Experiences regarding the online test modules

	Unassisted gastroenterologists (N = 6)			AI-assisted gastroenterologists (N = 6)		
	Test set 1	Test set 2	*P*-value	Test set 1	Test set 2	*P*-value
Quality of the images used in the test tests			0.574			0.545
• Poor	1 (16.7%)	1 (16.7%)		0	2 (33.3%)	
• Moderate	5 (83.3%)	4 (66.7%)		4 (66.7%)	3 (50.0%)	
• Good	0 (0%)	1 (16.7%)		2 (33.3%)	1 (16.7%)	
Difficulty rating of the test sets [0-10 scale]	7.1 (±1.5)	7.8 (±1.6)	0.478	6.3 (±2.3)	7.6 (±0.9)	0.215

As shown in Supplementary Table S9, unassisted gastroenterologists and AI-assisted gastroenterologists spent equal time to the test set before training (7.0 (±3.7) vs 9.6 (±2.9) minutes, *P*=0.237) and to the test set at follow-up (6.0 (±2.4) vs 7.5 (±1.4) minutes, *P*=0.209). The unassisted gastroenterologists spent less time to the post-training test than the AI-assisted gastroenterologists (6.7 (±2.4) vs 10.5 (±3.1) minutes, *P*=0.038). Lastly, the unassisted gastroenterologists spent equal time (*P*=0.185) to test set 2 when they retook the test with AI support after the cross-over (7.7 (±2.3) minutes) as they did previously without AI support (6.0 (±2.4) minutes) (*P*=0.185). Lastly, there were no differences in the time spent to the test sets at the various moments among both the unassisted (*P*=0.834) and the AI-assisted gastroenterologists (*P*=0.148).

In addition, the unassisted and AI-assisted gastroenterologists had equal intervals between the first test set and the second test set after training (25.5 (±11.3) vs 21.0 (±6.7) days, *P*=0.421), with a pooled average of 23.3 (±9.1) days for both groups.

Supplementary Table S9 Time spent to the two online test sets and the time between completing test sets 1B and 2.

	Unassisted gastroenterologists (N = 6)		AI-assisted gastroenterologists (N = 6)		*P*-value for comparison in time spent to test sets between unassisted and AI-assisted gastroenterologists
	Time spent (minutes)	*P*-value for comparison in group	Time spent (minutes)	*P*-value for comparison in group	
Pre-training test (test set 1A)	7.0 (±3.7)	0.834	9.6 (±2.9)	0.148	0.237
Post-training test (test set 1B)	6.7 (±2.4)		10.5 (±3.1)		0.038
Follow-up test (test set 2)	6.0 (±2.4)		7.5 (±1.4)		0.209

SUPPLEMENTARY MATERIAL S6: man-machine collaboration during the online modules

In Supplementary Figure S2, an overview of the interaction between gastroenterologists and AI as second assessor before training (A), after training (B), and at follow-up (C) can be found. The proportions of cases in which the gastroenterologists called for AI-assistance are presented by the bars in the middle. The man-machine interaction is represented by the proportions in which gastroenterologists accepted or rejected an AI diagnosis after calling for AI-assistance (graphs to the left) or assessed an EC image by themselves without calling for AI-assistance (graphs to the right).

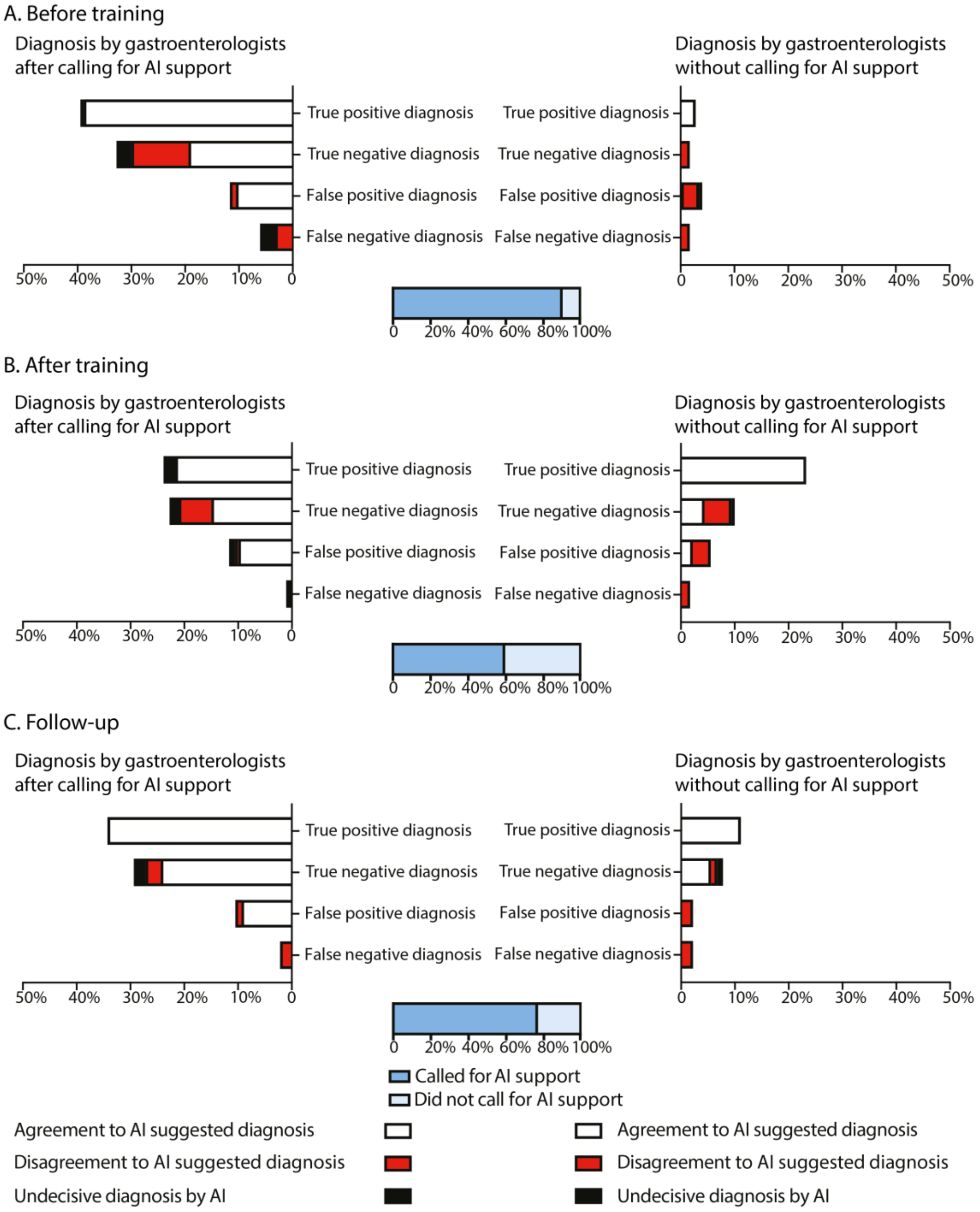

Supplementary Figure S2. Overview of the man-machine collaboration during the online modules.

REFERENCES

1. Pohl H, Koch M, Khalifa A, et al. Evaluation of endocytoscopy in the surveillance of patients with Barrett's esophagus. Endoscopy. 2007;39:492-6. doi:10.1055/s-2007-966340
2. Tomizawa Y, Iyer PG, Wongkeesong LM, et al. Assessment of the diagnostic performance and interobserver variability of endocytoscopy in Barrett's esophagus: a pilot ex-vivo study. World J Gastroenterol. 2013;19:8652-8. doi:10.3748/wjg.v19.i46.8652
3. Singh R, Yeap SP. Endoscopic imaging in Barrett's esophagus. Expert Rev Gastroenterol Hepatol. 2015;9:475-85. doi:10.1586/17474124.2015.983080
4. Pohl H, Rösch T, Vieth M, et al. Miniprobe confocal laser microscopy for the detection of invisible neoplasia in patients with Barrett's oesophagus. Gut. 2008;57:1648-53. doi:10.1136/gut.2008.157461
5. Sharma P, Meining AR, Coron E, et al. Real-time increased detection of neoplastic tissue in Barrett's esophagus with probe-based confocal laser endomicroscopy: final results of an international multicenter, prospective, randomized, controlled trial. Gastrointest Endosc. 2011;74:465-72. doi:10.1016/j.gie.2011.04.004
6. Tofteland N, Singh M, Gaddam S, et al. Evaluation of the updated confocal laser endomicroscopy criteria for Barrett's esophagus among gastrointestinal pathologists. Dis Esophagus. 2014;27:623-9. doi:10.1111/dote.12121
7. Kara MA, Ennahachi M, Fockens P, et al. Detection and classification of the mucosal and vascular patterns (mucosal morphology) in Barrett's esophagus by using narrow band imaging. Gastrointest Endosc. 2006;64:155-66. doi:10.1016/j.gie.2005.11.049
8. van der Wel MJ, Coleman HG, Bergman J, et al. Histopathologist features predictive of diagnostic concordance at expert level among a large international sample of pathologists diagnosing Barrett's dysplasia using digital pathology. Gut. 2020;69:811-22. doi:10.1136/gutjnl-2019-318985
